# Revealing Trapped Carrier Dynamics at Buried Interfaces in Perovskite Solar Cells via Infrared‐Modulated Action Spectroscopy with Surface Photovoltage Detection

**DOI:** 10.1002/adma.202502160

**Published:** 2025-04-11

**Authors:** Beier Hu, Tiankai Zhang, Longren Li, Haoqing Ning, Ganghong Min, Tong Wang, Mengyun Chen, Jiaxin Pan, Niansheng Xu, Thomas J. Macdonald, Feng Gao, Igal Levine, Ziming Chen, Artem A. Bakulin

**Affiliations:** ^1^ Department of Chemistry and Centre for Processable Electronics Imperial College London London W12 0BZ UK; ^2^ Department of Physics Chemistry and Biology (IFM) Linköping University Linköping SE‐58183 Sweden; ^3^ School of Materials Science and Engineering Southeast University Nanjing 211189 P. R. China; ^4^ Department of Electronic and Electrical Engineering University College London London WC1E 7JE UK; ^5^ Institute of Chemistry and The Center for Nanoscience and Nanotechnology The Hebrew University of Jerusalem Jerusalem 91904 Israel; ^6^ Department of Mechanical Engineering The University of Hong Kong Pokfulam Hong Kong 999077 P. R. China

**Keywords:** buried interface, perovskite solar cells, surface photovoltage, traps, ultrafast action spectroscopy

## Abstract

Interfacial engineering is a proven strategy to enhance the efficiency of perovskite solar cells (PeSCs) by controlling surface electronic defects and carrier trapping. The trap states at the “top” interface between the perovskite and upper charge extraction layers are experimentally accessible and have been extensively studied. However, the understanding of the unexposed “bottom” surface of the perovskite layer remains elusive, due to the lack of selective and non‐destructive tools to access buried interface. Here, a new spectroscopy technique is introduced that monitors nanosecond to millisecond dynamics of trapped carriers at the buried interfaces by combining optical trap activation by infrared light with surface photovoltage detection. Applied to various PeSC architectures, this method reveals that most interfacial traps reside between the perovskite and hole transport layer, suggesting a predominance of hole traps (e.g., cation and lead vacancies) over electron traps (e.g., halide vacancies) in the studied PeSC systems. The proposed new approach separates interfacial carrier‐loss contributions from the top and buried surfaces, providing design insights for achieving high‐performance PeSCs through interface optimization.

## Graphical abstract

A new spectroscopy technique (i.e., pump‐push‐surface photovoltage) is introduced that monitors trapped carrier dynamics at the buried interfaces in perovskite solar cells (PeSCs) via combining optical trap activation by infrared light with surface photovoltage detection. The proposed new approach separates interfacial carrier‐loss contributions from the top and buried surfaces, providing design insights for achieving high‐performance PeSCs through interface optimization.

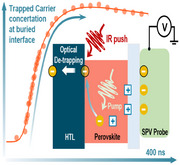

## 1 Introduction

Perovskite solar cells (PeSCs) have emerged as one of the most compelling contenders in photovoltaic technology, with the certified power conversion efficiency (PCE) recently surging to 27%.^[^ ^1^, ^2^ ^]^ Despite the unique defect tolerance nature of perovskites, a decent concentration of intragap electronic defect states forms during the solution‐processed fabrication, mainly accumulating at material surfaces and grain boundaries. They serve as trap states for photogenerated carriers, contributing to the unfavorable non‐radiative recombination and hence reducing the PeSC efficiency and stability.^[^ ^3^, ^4^, ^5^ ^]^

The formation of interfacial traps at perovskite top and bottom surfaces is related to various crystallization mechanisms (e.g., the top‐down growth). For instance, in the 3D perovskites, crystallization predominantly proceeds from the top to the bottom surface. This is driven by rapid solvent supersaturation at the top surface, which induces prompt crystal nucleation at the top followed by the downward crystal growth.^[^ ^6^, ^7^, ^8^, ^9^ ^]^ As a result, the top surface of perovskite film might bear numerous dangling chemical bonds; while the bottom surface is more likely to suffer from trap states due to the interrupted crystal growth, lattice mismatching, and strain.^[^ ^6^, ^7^, ^8^, ^9^ ^]^ These structural disruptions at both surfaces highlight the need for effective interfacial optimization strategies to inactivate surface traps in PeSCs. In the standard PeSC architectures, perovskite active layer is vertically sandwiched between charge transport layers (CTLs): hole‐transport layer (HTL) and electron‐transport layer (ETL). Varying the deposition sequences of functional layers enables the fabrication of PeSCs with conventional configuration (n‐i‐p) and inverted configuration (p‐i‐n), both of which exhibit promising and competitive potential in terms of photovoltaic performance.^[^ ^10^ ^]^ Properly modifying the interfaces between the perovskite and CTLs, which is widely achieved by the use of passivation agents, self‐assembled monolayers (SAMs), and functionalized CTLs, is crucial for suppressing trap‐assisted recombination, promoting charge collection, and thereby minimizing carrier loss.^[^ ^11^, ^12^, ^13^, ^14^, ^15^ ^]^

To date, the carrier trapping processes at the upper interface can be directly accessed by surface‐sensitive tools which provide feedback for the top CTL optimization.^[^ ^16^, ^17^ ^]^ At the same time, selectively discerning charge trapping properties and dynamical processes at the interface with the bottom CTL remains challenging. The difficulty arises from the buried nature of the bottom interface that complicates or even invalidates the direct use of surface‐sensitive characterization tools like X‐ray photoelectron spectroscopy and photothermal deflection spectroscopy.^[^ ^18^, ^19^, ^20^ ^]^ Specifically, the practical application of these conventional techniques addresses the necessity of an exposed surface, with the former being limited by the shallow penetration depth of X‐ray and the latter relying on surface‐localised heating modulation.^[^ ^21^, ^22^ ^]^ To expose and assess the buried surface, Zhu et al. conducted a direct investigation of traps at the bottom surface by peeling off the perovskite layer from the substrate.^[^ ^23^ ^]^ However, this is a sample‐destructive method that might induce extra dangling bonds during the peeling‐off process. Alternatively, the trap‐associated properties of the buried interface can be indirectly probed via the film quality of the upper perovskite layer, for instance based on its top‐view morphology or relevant charge dynamics.^[^ ^24^, ^25^, ^26^, ^27^ ^]^ The lack of the direct technique to probe the dynamics at buried interface nowadays calls for new characterisation approaches that allow for straightforward and non‐invasive observation of defects at the perovskite bottom surface.

Time‐resolved action spectroscopies are a rapidly growing field.^[^ ^28^, ^29^, ^30^, ^31^, ^32^, ^33^, ^34^ ^]^ The optical‐control method involving bound (e.g., exciton or trapped carrier) state generation by visible “pump” light followed by its re‐activation by infrared (IR) “push” light, has been shown to selectively address the dynamics of trapped carriers. Two alternative action‐detection approaches of this method have been reported so far, based on either detecting the IR‐induced photocurrent (i.e., pump‐push‐photocurrent, PP‐PC) or photoluminescence (pump‐push‐photoluminescence, PP‐PL).^[^ ^35^, ^36^, ^37^, ^38^ ^]^ While providing a unique insight into the trapped carrier dynamics, these methods are not directly suitable to selectively address buried interfaces. PP‐PC requires a complete device for the measurement, which means the detected signals are associated with charge trapping at both top and bottom perovskite‐CTL interfaces, as well as in the bulk of the active layer. The use of PP‐PL waives the need for the complete device, while maintaining the selectivity for bound/trapped states. However, it is more applicable to the systems where trapped states luminescence is strong and separable from other emissive processes, making PP‐PL less suitable for perovskite photovoltaics. Combining IR control with a selective action detection approach that makes the non‐radiative loss of individual interfaces detectable would open possibilities for new spectroscopic method to study the buried interfaces.

Surface photovoltage (SPV) spectroscopy has been broadly used in semiconductor research over the past 50 years to identify the presence of electronic defects based on the illumination‐induced charge separation.^[^ ^39^, ^40^ ^]^ It is an action‐detection technique which is applicable to both thin films and devices and has been demonstrated to reveal the trapping dynamics at the buried interface.^[^ ^41^ ^]^ Levine et al. investigated the transient SPV evolution under pulsed photoexcitation and presented a picture illustrating the combination of several charge dynamics processes, directly probing charge extraction to the different SAM‐based CTLs, while revealing electron trapping in the bottom interface of perovskite thin films.^[^ ^42^ ^]^ However, the transient SPV signal mainly arises from the redistribution of all the photoexcited carriers in the sample as a function of time, making it difficult to selectively probe the charge carrier dynamics of the trapped carriers only. Similarly, Chen et al. distinguished the ultrafast charge transfer and trapping dynamics based on the SPV transients ascribed to the photogenerated electrons and holes in photocatalyst, respectively. Nevertheless, the reliability of this assignment still needs to be enhanced with the temporal and spatial evidence obtained through pump‐probe photoemission electron microscopy.^[^ ^30^ ^]^

To tackle the limitation of aforementioned techniques and reveal the trapped carrier dynamics in the buried interface, a combination of IR optical trapped carrier activation and SPV detection can be a feasible approach, because: i) pump‐push mechanism is solely sensitive to bound states (e.g., traps) and non‐sensitive to free carriers, ii) SPV detection does not require a top electrode contact and hence allows the measurement of various types of samples involving a full device or half cells with sequentially deposited functional layers,^[^ ^41^, ^43^ ^]^ which enables the identification of the local characteristics at the individual interface.

Herein, we demonstrate a novel action spectroscopy technique (i.e., pump‐push‐SPV, PP‐SPV) that integrates the existing optical‐pump – IR‐push spectroscopy with the SPV detection scheme for probing carrier trapping dynamics at buried interfaces. Using this technique, we decode the trapped carrier dynamics including the timescales of trap filling and trap‐assisted recombination at the selected interfaces with nanosecond (ns) time resolution. We first compare the trapped carrier properties and dynamics in a full device obtained from traditional PP‐PC and newly developed PP‐SPV, confirming the validity of the approach. Then, by evaluating the quasi‐steady‐state PP‐SPV signal on both full devices and half cells (stacks of functional layers without metal electrode deposition), we find that a higher density of traps always presents at the perovskite/HTL interface, in both conventional and inverted cells. Using time‐resolved PP‐SPV, we are able to distinctly designate the trapped carrier dynamics at the buried interface in real‐time, by studying the ITO/ETL/Perovskite and ITO/HTL/Perovskite samples. We conclude that PeSCs with conventional and inverted structures indeed suffer from different charge trapping processes at top and bottom interfaces, which is further supported by a drift‐diffusion model. To mitigate the carrier loss in the inverted device, we passivate the defects at the bottom interface, resulting in a reduced number of trapped holes and therefore a shorter trap filling time. Our work presents the first report combining the SPV detection with pump‐push action spectroscopy, paving the way for the non‐destructive measurement of the trapped carrier dynamics at the buried interface of PeSCs.

## 2 Results and Discussion

**Figure 1:**
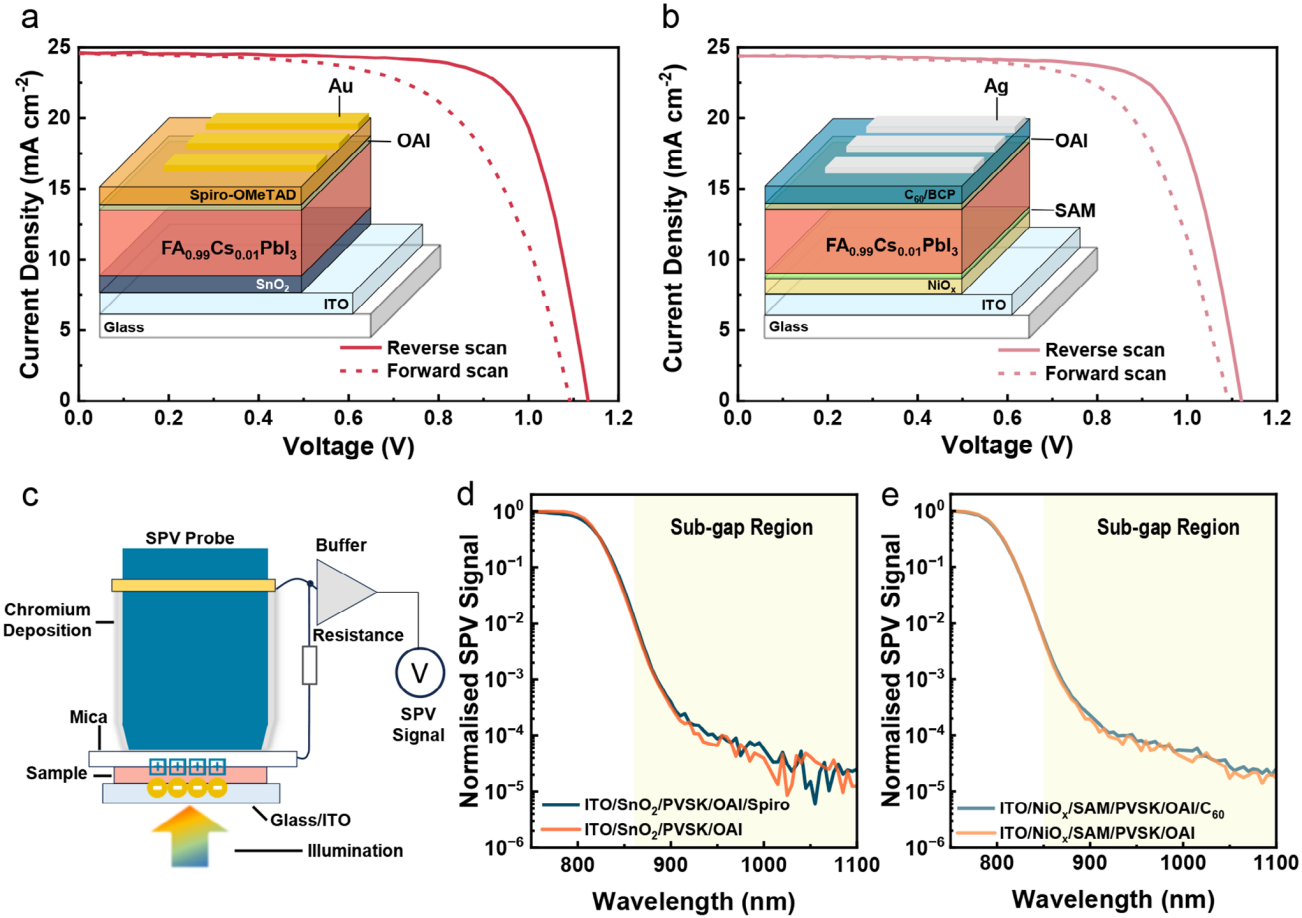
*J*–*V* curves of PeSCs with a) conventional and b) inverted structures. Insets in (a) and (b) show the corresponding device architectures. c) Schematic illustration of SPV measurement. Chromium is evaporated onto the surface of the SPV probe to establish the electrical connection. The light is incident from the glass side of the sample. Light‐modulated SPV spectra of half‐cell stacks with/without the top CTL in (d) conventional and (e) inverted architectures. Silicon Carbide lamp is used for providing light source, while monochromator is employed for wavelength selection.

Both conventional n‐i‐p and inverted p‐i‐n PeSCs were investigated in our research. The conventional PeSCs with an architecture of indium tin oxide (ITO)/tin oxide (SnO_2_)/FA_0.99_Cs_0.01_PbI_3_ (PVSK)/n‐octylammonium iodide (OAI)/2,2′,7,7′‐Tetrakis[N,N‐di(4‐methoxyphenyl)amino]‐9,9′‐spirobifluorene (Spiro‐OMeTAD, abbreviated as Spiro)/Au showed a decent PCE of 21.0% and a *V_oc_* of 1.13 V (**Figure** **1**a; Table S1, Supporting Information). The inverted devices were based on ITO/nickel oxide (NiO*_x_*)/2‐(9H‐carbazol‐9‐yl)ethyl)phosphonic acid (SAM)/PVSK/OAI/C_60_/Bathocuproine (BCP)/Ag architecture, achieving the PCE of 20.5% and a *V_oc_* of 1.12 V (Figure 1b; Table S1, Supporting Information). The OAI and SAM layers were used to passivate the surface defects of perovskite and NiO*_x_*, respectively.^[^ ^44^ ^]^

To identify the presence of sub‐gap states in the studied systems, we performed steady‐state light‐modulated SPV experiments on the perovskite active layer deposited with different combinations of CTLs.^[^ ^41^ ^]^ Samples were illuminated by a modulated monochromatic light from the glass side of the sample as shown in Figure 1c. Under the built‐in field, photogenerated electrons and holes diffuse to opposite surfaces of perovskite, inducing a new carrier distribution in the interfacial space‐charge layers. The induced charges on the top side polarise the mica sheet, which is subsequently detected using an SPV probe and a lock‐in amplifier. Figure 1d,e displays the SPV response involving the above‐ and below‐ bandgap excitation from the conventional and inverted samples with and without top CTL. All samples exhibit sub‐gap signals within the instrument sensitivity (Figure S1, Supporting Information), signifying the presence of trap states. However, the similarity of the SPV spectra across all four measurements indicates that, while quasi‐steady‐state SPV can probe defect states, it is not sufficiently/directly sensitive to the nature or location of the interface between the perovskite and the CTLs. Therefore, a more advanced and insightful experimental technique is required to elucidate the interface‐specific dynamics of trapped carriers.

**Figure 2:**
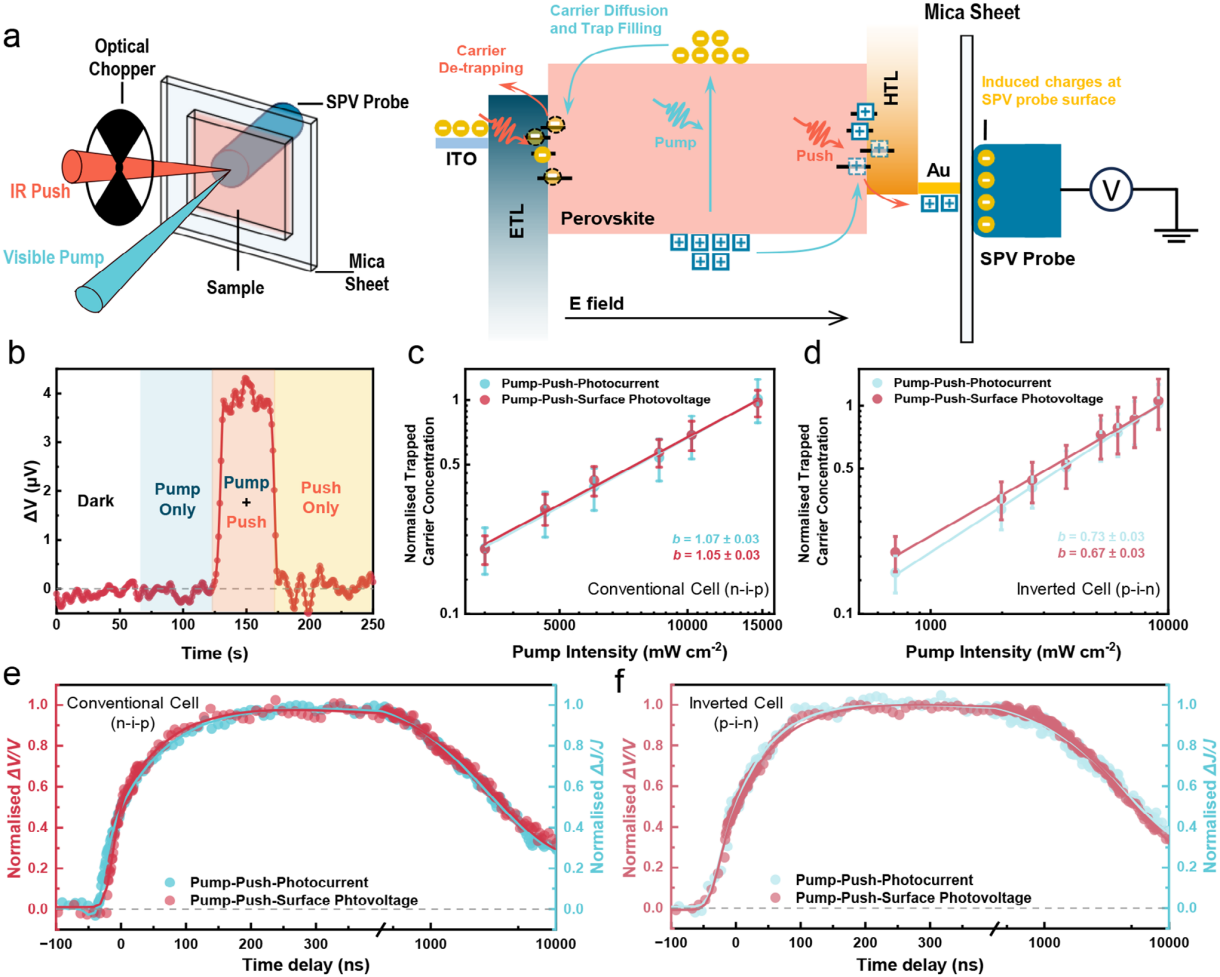
a) Conceptual diagram and working principle of the PP‐SPV setup. b) Illustration of pump‐push effect from a full n‐i‐p device observed by SPV detection. c) The concentration of trapped carriers as a function of pump intensity in a conventional PeSC (Pump: 450 nm; Push: 980 nm, 10 600 mW cm^‒2^). d) The concentration of trapped carriers as a function of pump intensity in an inverted PeSC (Pump: 450 nm; Push intensity: 20 800 mW cm^‒2^). e) Dynamics of charge trapping in a conventional device traced by PP‐PC and PP‐SPV (Pump: 515 nm; Push: 1064 nm, 1.34 mJ cm^‒2^). f) Dynamics of charge trapping in an inverted device traced by PP‐PC and PP‐SPV (Pump: 515 nm; Push: 1064 nm, 1.91 mJ cm^‒2^). To improve the signal‐to‐noise ratio, the transient PP‐SPV results shown in (e) and (f) are the averaged curves derived from pump‐fluence‐independent dynamics shown in Figures S6 and S7 (Supporting Information). The solid fitting lines are utilized as guides for eyes.

We therefore combined SPV detection with IR optical‐control approach which is intrinsically sensitive to bound states and can separate trapped carriers at different interfaces with varying sample structures. The PP‐SPV setup and its working principle are presented in **Figure** **2**a. Similar to traditional pump‐push techniques (e.g., PP‐PC): a visible (pump) beam is focused on the sample to create the population of free carriers.^[^ ^37^ ^]^ Photogeneration of carriers is followed by their trapping in the intra‐band electronic states located in the bulk of the material as well as at the material interfaces. Then a modulated IR (push) light is used to reactivate and release these trapped carriers, promoting them from intragap traps back to the conduction/valance band states in an energetic landscape, and other IR‐induced transitions have negligible impact (Figure S2; Note S1, Supporting Information). Once reactivated, these mobile carriers further conduct macroscopic charge separation, modulating carrier distribution in space‐charge region, hence generating additional SPV output. As PP‐SPV is only sensitive to trapped carriers, the signal (*ΔV*) is only produced when both visible pump (to create trapped carriers) and IR push (to reactivate trapped carriers) sources illuminate the sample simultaneously; a negligible signal is observed when only pump or push is on (Figure 2b). We emphasize that only the traps that are effective (those does not undergo thermal de‐trapping) and active (e.g., electron traps at ETL/PVSK interface, and hole traps at HTL/PVSK interface) in inducing substantial carrier loss are detectable in the pump‐push scheme, directly linking the microscopic defect to the macroscopic device performance.

To confirm the validity of PP‐SPV, we compared the results of PP‐PC measurement on the complete device to PP‐SPV measurement on the identical conditions. To simplify the comparison, we compared the trapped carrier concentration in both measurements, which was positively correlated to *ΔJ/J* in PP‐PC and *ΔV/V* in PP‐SPV experiments. We note that for *ΔV/V*, in the low‐signal approximation, it exhibits linear proportionality to the trapped carrier concentration (for more details see Note S2 and Figures S3–S5, Supporting Information). Figure 2c shows that the trapped carrier concentration (*n_TC_*) scales up as a power law with pump intensity (*n_TC_* ∝*I_pump_* ^*b*^) for both PP‐PC and PP‐SPV. The *b* value here reflects the occupation level of trap states, with *b *= 1 indicating high availability of trap states and *b *= 0 reflecting saturation and a full occupation of trap states.^[^ ^37^ ^]^ The extracted *b* values are close to 1 in both PP‐PC and PP‐SPV measurements, indicating that traps are far from being filled in the n‐i‐p device. The lower but still matching *b* values of ∼0.7 are observed in inverted PeSCs (Figure 2d) using both techniques, suggesting that a higher fraction of traps is filled (and overall few empty traps present) in inverted device architecture.

We also found time‐resolved PP‐SPV could track charge trapping dynamics. For this, we utilized pulsed lasers as pump and push sources, and measured PP‐SPV signal as a function of time delay between pump and push pulses. The dynamics of PP‐SPV and PP‐PC in PeSCs match well throughout the entire ns‐10 µs time window (Figure 2e,f; Figures S6 and S7; Note S3, Supporting Information). This agreement is consistent for both conventional and inverted devices. Both PP‐PC and PP‐SPV signals show instant component followed by <200 ns rise which we attribute to the bulk and interfacial trap filling, respectively, based on our previous work.^[^ ^37^ ^]^ The slow decay persisting over microseconds reflects the first‐order recombination of trapped carriers.^[^ ^45^ ^]^ Regardless of PeSCs structures, the perfect consistencies between these two detection approaches in quasi‐steady‐state and time‐resolved measurements suggest that PP‐SPV can act as robust probe for trapped carriers in devices alongside the recently developed PP‐PC spectroscopy. This also hints the nature of traps in PeSCs remains unchanged under both short‐circuit and open‐circuit conditions. Hence, we conclude that the SPV detection possesses comparable capability to photocurrent detection for observing trapped carriers in PeSC devices.

In contrast to PP‐PC measurements, which probe all trapped carriers throughout the entire device, PP‐SPV can isolate interfacial contributions when applied to the incomplete device stacks that lack one of the charge extraction layers. When a perovskite device includes two functional extraction layers, IR push photons reactivate trapped carriers at both interfaces, allowing them to travel under the built‐in electric field and produce a PP‐SPV response (Figure 2a). However, if one extraction layer is absent, the reactivated carriers at that interface remain localized at the interface and not extracted, resulting in negligible net charge redistribution. Consequently, the measured PP‐SPV signal stems primarily from the interface with a functional extraction layer (for more detailed discussion see Note S4; Figures S8–S10, Supporting Information). This approach thus enables PP‐SPV to selectively probe charge trapping and interfacial dynamics at individual buried interfaces—even (and especially) when the opposite side of the perovskite lacks a functional extraction contact.

**Figure 3:**
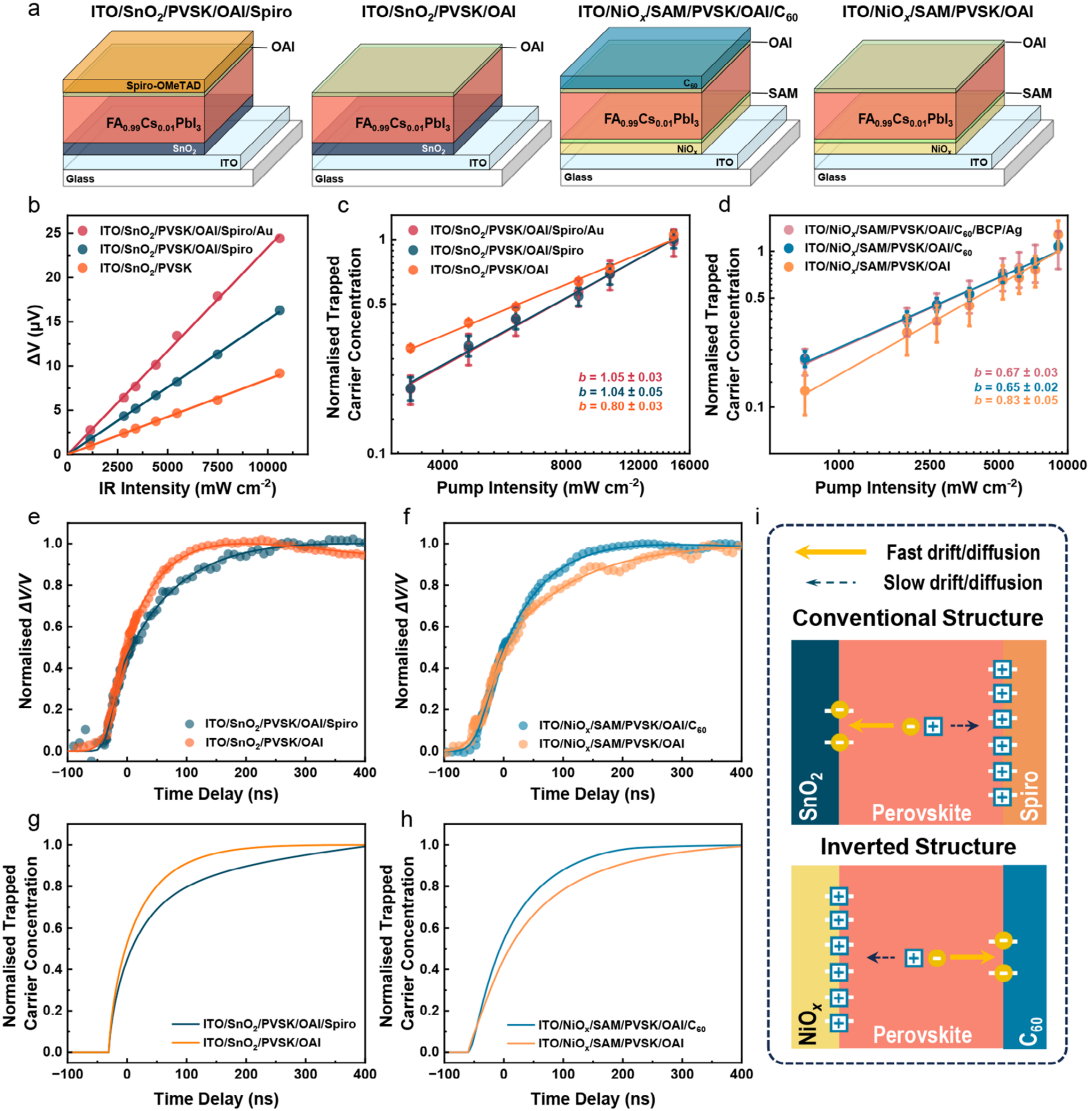
a) Perovskite half cells with different configurations. b) Linear growth of the IR‐induced SPV signals (*ΔV*) as increasing push intensity observed from samples with n‐i‐p structure. All data is fitted with linear equation (Pump intensity: 14 633 mW cm^‒2^). Normalized *n_TC_* as a function of pump intensity for whole/half devices with c) conventional configuration (Pump: 450 nm; Push: 980 nm, 10 600 mW cm^‒2^) and d) inverted configuration (Pump: 450 nm; Push: 980 nm, 20 800 mW cm^‒2^). The fitting lines in (c) and (d) are based on power law. Time‐resolved dynamics of the trapped carriers for (e) conventional half devices with and without the top Spiro layer (Pump: 515 nm; Push: 1064 nm, 1.91 mJ cm^‒2^), and f) inverted half devices with and without top C_60_ layer (Pump: 515 nm; Push: 1064 nm, 1.34 mJ cm^‒2^). To improve the signal‐to‐noise ratio, the transient PP‐SPV results in (e) and (f) are the averaged curves derived from pump‐fluence‐independent dynamics shown in Figures S16 and S19 (Supporting Information). The solid lines in (e) and (f) are used as guides for eyes. The trap filling time (*τ_rise_*), is defined as the time taken to reach 90% of the signal's full height. Concentration of trapped carriers at one/both interface(s) as the function of time in samples with different layers assembled as (g) conventional device configuration and (h) inverted device configuration, simulated by drift‐diffusion model. i) The schematic illustration of the correlation between interfacial trap distribution and interfacial trap filling dynamics in conventional/inverted cells.

To distinguish trap behavior at the perovskite bottom surface from that at both surfaces, we compared the PP‐SPV signal in the full devices and corresponding half devices with and without top CTL in both conventional (i.e., Spiro) and inverted (i.e., C_60_) configurations, as shown in **Figure** **3**a.^[^ ^44^, ^46^ ^]^ As presented in Figure 3b, the *ΔV* signals induced by the optically de‐trapped carriers scale up linearly with IR intensity for all sample structures. This indicates that the used IR intensity does not release enough carriers to disrupt the equilibrium between band‐edge carriers and trapped carriers, thus making IR excitation a suitable probe. Measuring pump intensity dependence of PP‐SPV in conventional samples with and without top HTL allows us to examine the saturation level of traps and gather insight into the interfacial defect density in both systems (Figures 3c; Figure S11, Supporting Information). The *b* value in ITO/SnO_2_/PVSK/OAI/Spiro is ∼1 and very similar to complete device, inferring a high concentration of available traps. This suggests that the deposition of top metal electrode, despite changing the built‐in field, has a minor macroscopic impact on the occupation of traps. In contrast, the ITO/SnO_2_/PVSK/OAI sample, without the top CTL layer, shows notably lower *b* of ∼0.8 which indicates lower concentration of detectable traps. Since only trapped carriers at the perovskite bottom PVSK/ETL interface are detectable in the latter case, we conclude that, in conventional n‐i‐p cell, the bottom interface exhibits a lower (electron) trap density, while the top surface with its higher (hole) trap density dominates the charge trapping.

We repeated the PP‐SPV power dependence experiments for inverted PeSCs to assess the trap density at the buried HTL/PVSK interface (Figure 3d; Figure S12, Supporting Information). Inverted device (ITO/NiO*_x_*/SAM/PVSK/OAI/C_60_/BCP/Ag) shows smaller *b* of ∼0.65, indicating reduced trap concentration. Similar as the conventional architecture, the *b* values obtained for complete device and ITO/NiO*_x_*/SAM/PVSK/OAI/C_60_ are consistent, confirming minor influence of top metal electrode. However, the sample without top ETL (ITO/NiO*_x_*/SAM/PVSK/OAI) shows a noticeably larger *b* value of 0.83, which is reflective of trapping at the bottom HTL/PVSK interface only. This result suggests that the trap density at the buried interface is greater than that in the top surface of perovskite. In summary, our findings indicate that regardless of the device architecture (conventional or inverted cells), a higher trap density can be always observed at the HTL/PVSK interface.

The dynamics of trapped carriers at the buried interface were further evaluated using time‐resolved PP‐SPV. Their recombination dynamics on the device‐performance‐relevant timescale (<10 µs) did not vary significantly among the samples (Figure S13, Supporting Information). We therefore focused on the early sub‐microsecond dynamics, which were attributed to interfacial trap filling (Figure 3e,f).^[^ ^37^ ^]^ When comparing devices with and without top metal electrode (Figure S14, Supporting Information), the rising time of PP‐SPV (τ*_rise_*) is notably decreased in the full device regardless of device configuration. Our previous study indicates the slow rise part of the signal corresponds to the drift/diffusion of free carriers to the interface, where they subsequently undergo the trap filling.^[^ ^37^ ^]^ Therefore, the observed reduction in *τ_rise_* reflects the accelerated drift/diffusion and can be attributed to the stronger built‐in field induced by the top metal electrode. To avoid the impact of the strong built‐in field in the whole device, we compared dynamics in film stacks without top metal electrode when disentangling trapping at perovskite's bottom and top surfaces.

For the conventional samples (Figure 3e), the faster *τ_rise_* of ≈80 ns in the sample without top HTL is delayed to ≈166 ns in sample with HTL, illustrating the faster trap filling process in the former (see also Figures S15 and S16, Supporting Information). This trend is confirmed across serval samples to rule out the film inhomogeneity (Figure S17, Supporting Information). We propose that high trap density at the top PVSK/HTL interface (revealed by quasi‐steady‐state PP‐SPV in Figure 3c), which localizes free holes, induces the formation of a strong positive interfacial charge layer during the trap filling process. The produced space charge layer screens the built‐in field and decelerates the drift of residual holes to the top interface.^[^ ^37^ ^]^ Accordingly, faster electron trap filling process occurs at the bottom ETL/PVSK surface due to the lower population of trapped electrons and the consequent formation of weaker interfacial charge layer. Therefore, in sample with top Spiro, where trapped carriers at both top and bottom surfaces are detectable, the overall charge trapping is governed by the slow hole trapping at the perovskite top surface due to the higher hole trap density. While in the sample without top Spiro layer where only trapped electrons in the perovskite bottom interface can be detected, a much faster rise of the signal is observed.

Conceptually, similar phenomena are observed in inverted cells (Figure 3f; Figures S18–S20, Supporting Information). The trap filling time in the ITO/NiO*_x_*/SAM/PVSK/OAI sample is ≈170 ns (*τ_rise_* solely for trapped holes at the perovskite bottom surface), much slower than ≈112 ns in the ITO/NiO*_x_*/SAM/PVSK/OAI/C_60_ sample where both HTL and ETL are preset. This suggests that drift/diffusion of holes to the bottom surface occurs slower than that of electrons to the top surface. It can also be attributed to the higher hole trap density at the bottom surface, where prevalent hole trapping forms a stronger interfacial charged layer that decelerates hole drift/diffusion. This conclusion also aligns well with the results of quasi‐steady‐state PP‐SPV measurements. For supporting the statement, space‐charge‐limited current measurement (SCLC), widely recognized as a feasible trap characterization approach, was conducted and confirmed the higher density of hole traps (2.8 × 10^15^ cm^‒3^) than the electron ones (2.6 × 10^15^ cm^‒3^) from the carrier‐only devices (Figure S21, Supporting Information). Compared to PP‐SPV, this less notable difference shown in SCLC could be attributed to the fact that the PP‐SPV selectively detects the interfacial traps effective and active in substantially triggering trap‐assisted loss, whereas SCLC estimates densities of overall electron or hole traps across the perovskites. It is also worth noting that the carrier mobilities in perovskite show order(s) of magnitude lower than that in all CTLs (Figure S21, Supporting Information), evidencing the dominant role of interfacial traps in the observed dynamics while ruling out the possibility of the delayed extraction due to insufficient mobilities of the much thinner CTLs.^[^ ^47^, ^48^, ^49^, ^50^ ^]^

To quantitatively verify the qualitative picture presented above, we applied a drift‐diffusion model to simulate the carrier dynamics and trapping. The model, apart from mobile carriers, included interfacial trap density, trap location, and trapped carrier dynamics in PeSCs with conventional and inverted structures. For direct comparison with experimental PP‐SPV data, the drift‐diffusion calculations evaluated the evolution of *n_TC_* at top/bottom interfaces over time (for details see Note S5; Table S2, Supporting Information). We found that the variations of the observed PP‐SPV kinetics could be reproduced just by varying the electron and hole trap density (*N_tn_* and *N_tp_*) at the designated perovskite top and bottom surfaces as an adjustment parameter. As shown in Figure 3g, for conventional samples, when the electron trap density at the bottom surface (1 × 10^15^ cm^‒3^) is set to half of the hole trap at the top surface of perovskite (2 × 10^15^ cm^‒3^), the simulated *n_TC_* growth in ETL‐only sample (only *n_TC_* at bottom interface) occurs substantially faster than that in the sample with both ETL and HTL (*n_TC_* in both bottom and top interfaces). Similarly, in inverted samples, when the hole trap density at the bottom surface (2 × 10^15^ cm^‒3^) is also set to twice higher than the electron trap density (1 × 10^15^ cm^‒3^), we notice a shorter *τ_rise_* in the ITO/NiO*_x_*/SAM/PVSK sample (only *n_TC_* at bottom interface), which speeds up further in presence of C_60_ layer (*n_TC_* in both bottom and top interface) (Figure 3h). These reproduced trends based on the drift‐diffusion model consolidate the conclusion that majority of traps are always located at the PVSK/HTL interface, irrespective of the device structure (Figure 3i).

**Figure 4:**
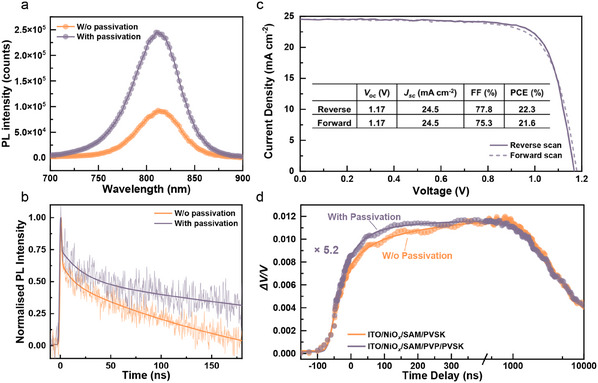
a) The steady‐state PL spectrum and b) Transient PL kinetics of perovskite films with and without bottom interface passivation (Glass/PVP/PVSK and Glass/PVSK, receptively). c) *J*–*V* characteristics of p‐i‐n device with buried interface passivation (ITO/NiO*_x_*/SAM/PVP/PVSK/OAI/C_60_/BCP/Ag). d) Dynamics of trapped carriers at bottom interface before and after interfacial passivation (ITO/NiO*_x_*/SAM/PVSK/OAI and ITO/NiO*_x_*/SAM/PVP/PVSK/OAI, respectively) (Pump: 515 nm, 28.9 uJ cm^‒2^; Push: 1064 nm, 2.10 mJ cm^‒2^). The amplitude of Δ*V*/*V* for passivated sample is multiplied by 5.2 times for better comparison. The solid lines in Figure 4b,d is used as guides for eyes.

After revealing that trap‐assisted losses preferentially happened at the buried interface in the inverted architecture, we passivated the bottom HTL/PVSK interface (NiO*_x_*/SAM/PVSK) by adding a thin layer of polyvinylpyrrolidone (PVP) between the HTL and PVSK and investigated the changes in the dynamics of trapped carriers. The primary role of PVP is confirmed as a defect passivator considering the unchanged top‐view morphology and crystallinity of perovskites (Figures S22 and S23, Supporting Information). The C═O group in the pyrrolidone unit can effectively passivate the perovskite surface by Lewis‐base‐Lewis‐acid interaction, proven by the enhanced PL intensity (**Figure** **4**a).^[^ ^51^ ^]^ Figure 4b displays the extended carrier lifetime in the PVP‐passivated sample, reaffirming the reduced non‐radiative recombination, which consequently enhances the device performance (Figure 4c) as reflected by the 0.05 V improvement in *V_oc_*.^[^ ^52^, ^53^ ^]^ The dynamics of trapped carriers at buried interface by PP‐SPV (Figure 4d) is also changed. The amplitude of *ΔV*/*V*, reflecting the concentration of trapped carriers, decreases by over fivefold after passivation. In addition, charge trapping mostly occurs within 65 ns in the PVP‐passivated sample, compared to 176 ns in non‐passivated one. This faster filling time of traps in the PVP‐passivated sample indicates a faster hole drift to the buried interface due to the weakened interfacial charge layer formed as a result of the reduced concentration of trapped holes between the PVP/PVSK interface.

## 3 Conclusion

In summary, we have reported a non‐invasive spectroscopic PP‐SPV approach to selectively observe charge trapping and trap‐mediated recombination at the buried interfaces in PeSCs. The matching PP‐SPV and PP‐PC responses validate the use of PP‐SPV as an *operando* probe for trapped carrier dynamics. We systematically investigated charge trapping occurring at the buried interface in samples with conventional and inverted configurations. In the conventional device, faster electron trap filling occurs at the SnO₂/PVSK interface, while hole traps at the PVSK/Spiro interface are filled at a slower rate. This delay is attributed to a repulsive force from the high concentration of trapped holes at the top interface, highlighting the dominant role of hole traps in the standard PeSC. Similarly, in the inverted counterparts, we identify that the primary trap‐assisted loss occurs at the buried interface, indicating that hole trapping near HTL side remains the major carrier loss pathway. Our results show that trap states at the PVSK/HTL interface consistently exhibit higher densities in both conventional and inverted devices. This suggests that our perovskite systems are more susceptible to forming hole traps (e.g., cation and lead vacancies) compared to electron traps (e.g., halide vacancies) which can be partly mitigated by treating interface with PVP. Our work demonstrates that PP‐SPV technique, capable of measuring stacks of films under operando conditions, can offer valuable insight into the design principles for PeSCs, particularly assisting and providing feedback for interfacial engineering. By enabling targeted interfacial optimization strategies for various perovskite systems, it is able to maximize the suppression of interfacial non‐radiative losses during layer‐by‐layer device fabrication, thereby enhancing the photovoltaic performance and operational stability, ultimately accelerating the large‐scale commercialisation of PeSCs.

## 4 Experimental Section

### Precursor Preparation

The FA_0.99_Cs_0.01_PbI_3_ perovskite precursor was prepared by dissolving 1.81 m lead iodide (PbI_2_) (Anhydro Beads, 99.999% Sigma‐Aldrich), 1.65 m formamidium iodide (FAI) (Greatcell solar), 0.58 m methylamonium choloride (MACl) (Xi'an Yuri Solar Corp.), 0.016 m Cesium iodide (CsI) (99.999% Sigma‐Aldrich) in the 1 mL mixed solvent of anhydrous N, N‐dimethylformamide (DMF, 99.8%, Sigma‐Aldrich) and dimethyl sulfoxide (DMSO, 99.9%, Sigma‐Aldrich) in a volume ratio of 8:1. The IM radical doping of Spiro‐OMeTAD (Xi'an Yuri Solar Corp.) was prepared by dissolving 90 mg mL⁻^1^ Spiro‐OMeTAD in chlorobenzene, followed by the addition of 6 mol% Spiro‐OMeTAD^2^⁺(TFSI⁻)^2^ and 18 mol% TBMP⁺TFSI⁻ (self‐synthesized).^[^ ^44^ ^]^

### Device Fabrication

n‐i‐p structure device: ITO substrates were first cleaned in deionized water and then in ethanol, each for 15 min. After drying, the substrates underwent a 15‐min UV‐ozone treatment. The SnO_2_ ETL was prepared by spin‐coating a 1:6 diluted SnO_2_ nanoparticle water solution (Alfa Aeser) at 4000 rpm for 30 s, and the film was annealed at 150 °C for 20 min in air. The perovskite active layer was deposited by spin‐coating the precursor at 5000 rpm for 30 s, with 100 µL of chlorobenzene dropped as an antisolvent at the 10^th^ s, after which the perovskite film was then annealed at 150 °C for 15 min in ambient air. To passivate the top surface, 5 mg ml^‒1^ n‐Octylammonium iodide (OAI, Yuri solar) solution (dissolved in tert‐butanol, anhydrous, 99.5%, Sigma‐Aldrich) was deposited onto the perovskite surface by spin‐coating at 5000 rpm and annealed at 100 °C for 3 min. The HTL, composed of ion‐modulated radical‐doped Spiro‐OMeTAD, was subsequently spin‐coated at 5000 rpm for 30 s without further annealing. An 80‐nm gold electrode was then deposited by thermal evaporation under high vacuum (pressure < 3 × 10⁻⁶ Torr). A shadow mask with an area of 0.06 cm^2^ was used to define the effective active area of the PeSCs.

p‐i‐n structure device: ITO substrates were first cleaned in deionized water and then in ethanol, each for 15 min. After drying, the substrates underwent a 15‐min UV‐ozone treatment. The NiO*_x_* HTL was prepared by spin‐coating a 1:5 diluted NiO*_x_* nanoparticle ethanol solution (Avanta) at 4000 rpm for 30 s, and the film was annealed at 150 °C for 20 min in air. A 0.4 mg mL⁻^1^ 2‐(9H‐carbazol‐9‐yl)ethyl)phosphonic acid (2PACz) solution (dissolved in ethanol) was spin‐coated onto the NiO*_x_* layer, followed by annealing at 100 °C for 10 min in the N_2_ glovebox. For the buried‐interface‐passivated sample, polyvinyl pyrrolidone (PVP, molecular weight ≈40 000) was dissolved in chlorobenzene at a concentration of 1 mg mL⁻^1^ and stirred for 6 hours before use at room temperature. The PVP passivation layer was then deposited by spin coating the PVP solution at 4000 rpm on top of the 2PACz and annealed at 100 °C for 10 min in the N_2_ glovebox. The perovskite active layer was deposited by spin‐coating the precursor at 5000 rpm for 30 s, with 100 µL of chlorobenzene dropped as an antisolvent at the 10^th^ s, after which the perovskite film was then annealed at 150 °C for 15 min in ambient air. To passivate the top surface, 5 mg ml^‒1^ n‐Octylammonium iodide (OAI, Yuri solar) solution (dissolved in tert‐butanol, anhydrous, 99.5%, Sigma‐Aldrich) was deposited onto the perovskite surface by spin‐coating at 5000 rpm and annealed at 100 °C for 3 min. The ETL (C_60_, 25 nm), buffer layer (BCP, 3 nm) and the electrode (Ag, 100 nm) were subsequently deposited by thermal evaporation under high vacuum (pressure < 3 × 10⁻^6^ Torr). A shadow mask with an area of 0.06 cm^2^ was used to define the effective active area of the PeSCs.

### Characterization

*J–V* measurements were performed using a source meter (Keithley 2400) under simulated AM 1.5G solar illumination (100 mW cm⁻^2^) irradiated by the sun simulator (AAA class, Enlitech). The light intensity was calibrated using a certified silicon reference cell. Devices had an active area of 0.06 cm^2^ defined by the shadow mask. *J–V* curves were recorded in both forward (–0.2 to 1.2 V) and reverse (1.2 to –0.2 V) directions, using a constant scan rate of 200 mV s⁻^1^. SCLC measurements were conducted on the hole‐only device (with the structure of ITO/NiO*_x_*/2PACz/PVSK/Spiro‐OMeTAD/Au) and the electron‐only device (with the structure of ITO/SnO_2_/PVSK/C_60_/BCP/Ag), respectively. The scan range is from 0 to 5 V and the scan rate is 500 mV s^‒1^. Silicon Carbide lamp (SLS203F, THORLABs) is focused into a 4 mm diameter beam spot to illuminate the samples from the glass side in the light‐modulated SPV measurement. The samples are placed in the home‐made SPV box as depicted in Figure 1c. The monochromatic light split by monochromator (MS257^TM^ 1/4 m, Newport) is under modulation of optical chopper (MC1F2, THORLABs) at 128 Hz. The SPV responses of samples under the excitation of the modulated light are then fed into a lock‐in amplifier (MFLI 500 kHz, Zurich Instruments), during which a 750‐nm long‐pass filter (FELH750, THORLABs) is mounted in front of samples to avoid the artifact from high‐order diffraction. PL spectra and time‐resolved PL data were measured at room temperature using a commercial system (FLS 1000, Edinburgh Instrument). The PL was collected under the excitation of the monochromatic light at 465 nm spectrograph from the Xenon lamp (100 mW cm⁻^2^), and the time‐resolved PL probed ≈810 nm was collected under the excitation of a picosecond 635‐nm pulsed laser source (time duration less than 100 ps, 0.1 MHz). X‐ray diffraction measurements were carried out using a Bruker D2 PHASER diffractometer equipped with the Cu Kα radiation source (λ = 1.5406 Å) operated at 40 kV and 40 mA. Scanning electron microscopic images were collected on a Zeiss Gemini 360 Scanning Electron Microscope operated at 3 kV using secondary electron image mode.

### Pump‐Push Spectroscopy

In quasi‐steady‐state pump‐push measurement, two continuous‐wave‐diode lasers are used as “pump” (CPS450, THORLABs) and “push” (CPS980, THORLABs) lasers, respectively. The “pump” and “push”, which are combined at a dichroic mirror (DMLP425, THORLABs), are overlapped and focused on the sample with the beam diameter ≈0.3 mm. The “push” beam is chopped at 717 Hz by an optical chopper (MC1F30, THORLABs) which is synchronised with the lock‐in amplifier (SR830, Stanford Research Systems). The pump‐push signal, which originates from the difference between “push” on and off controlled by the chopper, is thus collected by the lock‐in amplifier. Similarly, the chopper is placed in “pump” path when collecting the pump‐induced signal.

In time‐resolved pump‐push measurement, the “pump” (515 nm) is provided by the second harmonic generation of the Yb:KGW laser (Pharos, Light Conversion), while the “push” (1064 nm) is generated from Nd:YVO4 laser (Piccolo, Innolas Laser) with 15 ns time resolution which is synchronised with the fundamental Yb:KGW laser. The repetition rate of the system is divided by three times (3.33 kHz) from the default 10 kHz using pulse picker. The delay time of “pump” and “push” is controlled by an electrical delay generator (DG645, Stanford Research Systems). Analogous to quasi‐steady‐state pump‐push setup, the “pump” and “push” are aligned colinearly and then focused on the sample, while the “push” is modulated at 717 Hz by the chopper. Both the reference frequencies of “pump” (3.33 kHz) and “push” (717 Hz) are coupled to a two‐channel lock‐in amplifier so that pump‐induced and pump‐push signals can be identified separately. The pulse energies used in quasi‐steady‐state and time‐resolved PP‐PC and PP‐SPV measurements could be found in figure captions or legends. For SPV detection measurement, the sample (could be either device or films) is placed in the home‐made SPV box based on the parallel plate capacitor configuration, where an insulating mica sheet is sandwiched between the sample surface and SPV probe. For PC detection measurement, sample (must be full device) operating at short‐circuit condition is accommodated in custom‐made device holder.

## Conflict of Interest

The authors declare no conflict of interest.

## Supporting information



Supporting Information

## Data Availability

The data that support the findings of this study are available from the corresponding author upon reasonable request.
