# Supplementary material for: Revealing Trapped Carrier Dynamics at Buried Interfaces in Perovskite Solar Cells via Infrared‐Modulated Action Spectroscopy with Surface Photovoltage Detection
Source: Adv Mater. 2025 Apr 11;37(26):2502160. doi: 10.1002/adma.202502160 (PMC12232217; doi:10.1002/adma.202502160)
Supplement: Supplementary file 1 — Supporting Information [file ADMA-37-2502160-s001.docx]

**Supplementary Information**

for

**Revealing Trapped Carrier Dynamics at Buried Interfaces in Perovskite Solar Cells via Infrared-Modulated Action Spectroscopy with Surface Photovoltage Detection**

Beier Hu^1^, Tiankai Zhang^2,3^, Longren Li^1^, Haoqing Ning^1^, Ganghong Min^4^, Tong Wang^1^, Mengyun Chen^2^, Jiaxin Pan^1^, Niansheng Xu^2^, Thomas J. Macdonald^4^, Feng Gao^2^, Igal Levine^5,*^, Ziming Chen^1,6,*^, Artem A. Bakulin^1^

^1^ Department of Chemistry and Centre for Processable Electronics, Imperial College London, London W12 0BZ, United Kingdom

^2^ Department of Physics, Chemistry and Biology (IFM), Linköping University, Linköping, SE-58183, Sweden

^3^ School of Materials Science and Engineering, Southeast University, Nanjing, 211189, P.R.China

^4^ Department of Electronic and Electrical Engineering, University College London, London, WC1E 7JE, United Kingdom

^5^ Institute of Chemistry and The Center for Nanoscience and Nanotechnology, The Hebrew University of Jerusalem, Jerusalem, 91904, Israel

^6^ Department of Mechanical Engineering, The University of Hong Kong, Pokfulam, Hong Kong, 999077, China

Correspondence: Igal Levine (igal.levine@mail.huji.ac.il); Ziming Chen (z.chen@imperial.ac.uk); Artem A. Bakulin (a.bakulin@imperial.ac.uk).

**Figure S1.** **Steady-state SPV signal obtained for perovskite films with different half-cell configuration.** The sub-gap signals are one order of magnitude higher than the noise level.

**Supplementary Note 1: Working principle of pump-push effect**

**Figure S2. Schematic energy diagram of pump-push effect.** (a) Possible electronic transitions under ‘pump’ beam. (b) Possible electronic transitions under ‘push’ beam. Process (1) represents the optical de-trapping, process (2) depicts the optical excitation of band-edge carriers to hot states, and process (3) reflects the ultrafast hot carrier cooling.

Ideally, in pump-push-based action spectroscopy, a visible beam above the material’s bandgap (‘pump’) excites carriers, while an IR beam with sub-bandgap photon energy (‘push’) selectively reactivates bound species (e.g., trapped carriers). After excitation by ‘pump’, the photogenerated carriers produce pump-induced signals, symbolised as *V* or *J*, when some are captured by intra-band trap states (Figure S2a). The IR push then is expected to release these localised carriers back to the conduction or valence band energetically [Figure S2b, process (1)]. Since only the ‘push’ is under modulation at specific frequency by an optical chopper, the additional output induced by the arrival of IR as pump-push signals expressed as $\Delta V$ or $\Delta J$ could be identified and ultimately extracted by a lock-in amplifier. Note that since voltage or current differences are recorded, only trapped carriers undergoing recombination can be observed (contributing to $\Delta V$ or $\Delta J$). Carriers that are thermally de-trapped remain undetectable, as they ultimately contribute to $V$ or $J$, within the measurement time window.

Additionally, the dissociation of exciton by the IR push would be another possible transition to generate pump-push signal. However, the low exciton binding energy of 3D perovskite ensures the negligible population of exciton, making trapped carriers the sole bound state giving rise to the pump-push effect.^[1]^

Besides, we note that the IR can also optically excite the band-edge carriers to hot states [process (2) in Figure S2b]. However, due to their ultrafast relaxation [within few picoseconds, process (3) in Figure S2b], hot carrier is also considered not to contribute to the collected pump-push signal ($\Delta V$ or $\Delta J$) because of the following two reasons.^[2]^

First, theoretically, this process doesn’t change the number of band-edge carriers with and without IR push, as the hot carriers always cool down to become cold carriers eventually. Specifically, the signal of IR-induced hot carrier could be symbolised as $V_{hot-carrier}$, while the signal of band-edge cold carrier ($V_{cold-carrier}$) in the perovskite after IR push could be expressed as ($V_{pump}-V_{hot-carrier}$). Therefore, these two IR-induced processes cancel each other out, leaving the population of band-edge carriers unaffected by IR push. The overall IR-induced pump-push signal, attributed solely to trapped carriers, can be expressed as the SPV or photocurrent signal difference with and without the presence of ‘push’:

$$\Delta V=V_{pump+push}-V_{pump}=(V_{cold-carrier}+V_{hot-carrier}+V_{trapped-carrier})-V_{pump}=\left( \left( V_{pump}-V_{hot-carrier} \right)+V_{hot-carrier}+V_{trapped-carrier} \right)-V_{pump}$$

$=V_{trapped-carrier} \left( S1 \right)$

or

$$\Delta J=J_{pump+push}-J_{pump}=(J_{cold-carrier}+J_{hot-carrier}+J_{trapped-carrier})-J_{pump}=\left( \left( J_{pump}-J_{hot-carrier} \right)+J_{hot-carrier}+J_{trapped-carrier} \right)-J_{pump}$$

$=J_{trapped-carrier} \left( S2 \right)$

Second, practically, considering the relatively slow decay of band-edge carriers by bimolecular recombination, the perturbation of band-edge carrier population (via hot carrier formation and cooling) within few picoseconds still has a negligible impact on their bimolecular recombination change.

**Supplementary Note 2: Calculation of trapped carrier concentration**

**Figure S3.** **Comparison of pump-induced SPV and photocurrent as a function of pump intensity in (a) conventional and (b) inverted PeSCs.**

**Figure S4. The calculated *ΔV/V* in (a) ITO/SnO_2_/PVSK/Spiro/Au, (b) ITO/SnO_2_/PVSK/Spiro, and (c) ITO/SnO_2_/PVSK under different pump and push intensities.**

The intrinsic selectivity to the bound state of pump-push technique makes it possible to access the concentration of trapped carriers. The pump-induced signal (*V* or *J* in Figure S3) originates from the number of free carriers in material system, while the pump-push signal ($\Delta V$ or $\Delta J$) originates from the number of carriers de-trapped by the IR-reactivation.

In the case of SPV, the *V* can be expressed by^[3]^:

$$V=\frac{{I_{Pump}\cdot\lambda}_{Pump}}{hc}\cdot\left( 1-e^{{-\alpha}_{Pump}\cdot d} \right)\cdot E\left( I_{Pump} \right)\cdot k\left( I_{Pump} \right)\cdot A_{Q\to V} (S3)$$

where $\lambda_{Pump}$ and $\alpha_{Pump}$ are pump wavelength and corresponding absorption coefficient, respectively. Note that the intrinsic absorption coefficients of the 450-nm pump and 980-nm push beams for FAPbI_3_, which are reported from literatures, are 1×10^5^ cm^‒1^ and << 1 m^‒1^, respectively;^[4]^ *h* is Planck's constant; $c$ is the speed of light; $d$ is set to 1600 nm, which is twice of the thickness of the perovskite layer (800 nm) when considering light reflection by the electrode or SPV probe; $E\left( I_{Pump} \right)$ is the effective charge accumulation factor describing the level of charges that can accumulate at both surfaces, originated from the population of initial pump-induced charges; $k\left( I_{Pump} \right)$ describes the effective ability of inducing charges in the SPV probe originated from the electric field established by a specific spatial distribution of electrons and holes in the sample, generated by the pump beam; $A_{Q\to V}$ is the induced charges to voltage conversion factor, which is a constant as the *V* should be proportional to the amount of induced charges. $E\left( I_{Pump} \right)$ is a function of pump intensity ($I_{Pump}$) because: i) the dominant recombination process might change (i.e., first-order trap-mediated in bulk or at interface, band-to-band, or Auger recombination) when varying the pump intensity; ii) the diffusion of residual charges could be suppressed by the already accumulated charges at the surface; iii) the drift of residual charges could be suppressed by the screening of built-in field by the accumulated charges at the surface.

Similarly, we can also evaluate $\Delta V$ by the following expression:

$$\Delta V=\frac{{I_{IR}\cdot\lambda}_{IR}}{hc}\cdot\left( 1-e^{{-\alpha}_{IR}\cdot d} \right)\cdot E\left( I_{Pump},I_{Push} \right)\cdot k\left( I_{Pump},I_{Push} \right){\cdot A}_{Q\to V} (S4)$$

where $I_{IR}$ is the push intensity before chopper; $\lambda_{IR}$ is the push wavelength; $\alpha_{IR}$ is the absorption coefficient of trapped carriers which depends on the number of trapped carriers present$; E\left( I_{Pump},I_{Push} \right)$ is the effective charge accumulation factor describing the level of charges that can accumulate at both surfaces originated from the population of initial pump-induced charges and subsequent push-induced charges; $k\left( I_{Pump},I_{Push} \right)$ describes the effective ability of inducing charges in the SPV probe originated from the electric field established by a specific spatial distribution of electrons and holes in the sample, generated by both the pump and push beam.

Combining Equations S3 and S4, we can calculate $\Delta V/V$ as:

$$\frac{\Delta V}{V}=\frac{\frac{{I_{IR}\cdot\lambda}_{IR}}{hc}\cdot\left( 1-e^{{-\alpha}_{IR}\cdot d} \right)\cdot E\left( I_{Pump},I_{Push} \right)\cdot k\left( I_{Pump},I_{Push} \right){\cdot A}_{Q\to V}}{\frac{{I_{Pump}\cdot\lambda}_{Pump}}{hc}\cdot\left( 1-e^{{-\alpha}_{Pump}\cdot d} \right)\cdot E\left( I_{Pump} \right)\cdot k\left( I_{Pump} \right){\cdot A}_{Q\to V}}$$

$$\begin{aligned} =\frac{\left( 1-e^{-\sigma{\cdot n}_{TC}\cdot d} \right){\cdot\lambda}_{IR}\cdot E\left( I_{Pump},I_{Push} \right)\cdot k\left( I_{Pump},I_{Push} \right)}{\left( 1-e^{-\alpha_{Pump}\cdot d} \right)\cdot{I_{Pump}\cdot\lambda}_{Pump}\cdot E\left( I_{Pump} \right)\cdot k\left( I_{Pump} \right)}\cdot I_{IR}\#\left( S5 \right) \end{aligned}$$

where $\sigma$ is the absorption cross-section of trapped carriers and $\alpha_{IR}=\sigma\cdot n_{TC}$, while $n_{TC}$ refers to the trapped carrier concentration.^[3,5]^ Here, $\sigma$ originates from the intrinsic properties of trapped carriers, which should solely relate to the types of traps. In such a case, we consider $\sigma$, at a specific push wavelength, to be a constant. Therefore, we can simplify the discussion via the relationship of $\alpha_{IR}\propto n_{TC}$.

In PP-SPV, the charge accumulation factor of $E\left( I_{Pump},I_{Push} \right)$ is similar to $E\left( I_{Pump} \right)$ as the increased population of band-edge carriers generated by the IR push beam is small compared to the population of band-edge carriers generated by the pump beam. Therefore, the charge accumulation factor in the “pump only” and “pump + push” cases is very similar and makes $E\left( I_{Pump},I_{Push} \right)$/$E\left( I_{Pump} \right)\approx1$. Similarly, the spatial redistribution of electrons and holes induced by the IR push beam have a negligible impact on the overall spatial distribution of electrons and holes generated by the pump beams, which also makes $k\left( I_{Pump},I_{Push} \right)$/$k\left( I_{Pump} \right)\approx1$.

With these approximations, Equation S5 can be simplified as:

$$\begin{aligned} \frac{\Delta V}{V}=\frac{\left( 1-e^{-\sigma{\cdot n}_{TC}\cdot d} \right){\cdot\lambda}_{IR}}{\left( 1-e^{-\alpha_{Pump}\cdot d} \right)\cdot{I_{Pump}\cdot\lambda}_{Pump}}\cdot I_{IR}\#\left( S6 \right) \end{aligned}$$

Based on Equation S6, we see that $\Delta V/V$ depends linearly on $I_{IR}$ if the fractional changes in $n_{TC}$ induced by $I_{IR}$ are small (i.e., the low-signal scenario). This assumption is consistent with the results shown in Figures S4, where the data obtained from conventional samples is used as examples. In such a case, the slope of ($\Delta V/V$) *vs* $I_{IR}$ can be expressed and quantitatively extracted by the following equation:

$$\begin{aligned} \frac{d\left( \Delta V/V \right)}{dI_{IR}}=\frac{\left( 1-e^{-\sigma{\cdot n}_{TC}\cdot d} \right){\cdot\lambda}_{IR}}{\left( 1-e^{-\alpha_{Pump}\cdot d} \right)\cdot{I_{Pump}\cdot\lambda}_{Pump}}\#\left( S7 \right) \end{aligned}$$

Therefore, $n_{TC}$ can be expressed as a function of $I_{Pump}$ (as plotted in Figure S5):

$$\begin{aligned} n_{TC}\propto\alpha_{IR}=-\frac{1}{d}\cdot ln\{1-\frac{\left( 1-e^{-\alpha_{Pump}\cdot d} \right){\cdot\lambda}_{Pump}}{\lambda_{IR}}\cdot\frac{d\left( \Delta V/V \right)}{dI_{IR}}\cdot I_{Pump}\}\#\left( S8 \right) \end{aligned}$$

where the slope term can be determined from the relevant ($\Delta V/V$) *vs* $I_{IR}$ dependence measured experimentally. In low-signal scenarios, Equation S8 can be further approximated mathematically as:

$$\begin{aligned} n_{TC}\propto\alpha_{IR}=\frac{1}{d}\cdot\frac{\left( 1-e^{-\alpha_{Pump}\cdot d} \right){\cdot\lambda}_{Pump}}{\lambda_{IR}}\cdot\frac{d\left( \Delta V/V \right)}{dI_{IR}}\cdot I_{Pump}\#\left( S9 \right) \end{aligned}$$

making $n_{TC}$ linearly proportional to $\Delta V/V$ under specific $I_{Pump}$ and $I_{IR}$.

Similarly, in the case of the well-developed PP-PC,^[3]^ the $n_{TC}$ could be also written as a function of $I_{Pump}$:

$$\begin{aligned} n_{TC}\propto\alpha_{IR}=-\frac{1}{d}\cdot ln\{1-\frac{\left( 1-e^{-\alpha_{Pump}\cdot d} \right){\cdot\lambda}_{Pump}}{\lambda_{IR}}\cdot\frac{d\left( \Delta J/J \right)}{dI_{IR}}\cdot I_{Pump}\}\#\left( S10 \right) \end{aligned}$$


**Figure S5.** **Comparison for the trapped carrier absorption coefficient as a function of pump power in (a) conventional and (b) inverted PeSCs obtained from SPV detection and photocurrent detection.**

**Supplementary Note 3: Mathematical model for fitting pump-push transients**

The pump-push transients are all mathematically fitted by the exponential decay/rise function convoluted with Gaussian function as described in Equation S11:

$F\left( x \right)= \int_{-\infty}^{+\infty} \left\{ \frac{e^{-\frac{t^{2}}{2\sigma^{2}}}}{\sigma\sqrt{2\pi}}\times\left[ e^{-\frac{\left( x-x_{0} \right)-t}{Dec \tau_{i}}}\times(1-e^{-\frac{\left( x-x_{0} \right)-t}{Ing \tau_{i}}}) \right] \right\}\partial t (S11)$

where $\sigma$ represents width of the Gaussian component determined by time resolution (15 ns in this case), $x_{0}$ corresponds to the time zero, $Dec \tau_{i}$ and $Ing \tau_{i}$ symbolise the exponential time constants of decay components and growth components, respectively.^[6]^ We note that the PP-PC and PP-SPV dynamics are only mathematically fitted as a visual guide, without an underlying physical model.

**Figure S6.** **Trapped carrier dynamics in conventional PeSCs measured by PP-PC and PP-SPV.** Pump fluence dependence of the transient PP-PC signals (a) before and (b) after normalisation. Pump fluence dependence of transient PP-SPV signals (c) before and (d) after normalisation. The fluence-independent trapped carrier dynamics suggests that the charge trapping-relevant processes are dominated by first-order procedures. It hints that there is no energy exchange or interaction among trap states so that trapped carriers in whole conventional device behave individually without higher-order interaction (e.g. trapped carrier annihilation) under the presented excitation conditions.

**Figure S7.** **Trapped carrier dynamics in inverted PeSCs measured by PP-PC and PP-SPV.** Pump fluence dependence of the transient PP-PC signals (a) before and (b) after normalisation. Pump fluence dependence of transient PP-SPV signals (c) before and (d) after normalisation.

**Supplementary Note 4: Origin of PP-SPV signal**

**Figure S8. Origin of SPV signals with/without IR ‘push’ effect.** The equilibrium carrier distribution under photoexcitation in (a) defect-free and (b) defective perovskite without ‘push’ beam. (c) The carrier redistribution in defective perovskite in the presence of ‘push’ beam. The induced charges are contributed by the distribution of both electrons and holes in the perovskite. The ‘push’ beam is represented by the yellow arrow.

**Figure S9. Schematic illustration demonstrating the selectivity of the PP-SPV technique for the buried interface.** Only the pump-push effect in half-cell assembled as conventional PeSC structure is shown here for simplification. The ‘pump’ and ‘push’ beams are differentiated by blue and yellow arrows, respectively.

The SPV signal under ‘pump’ only excitation regulated as *V* is supposed to reflect the number of pump-induced charges in the sample. According to Equation S3, the amplitude of signal *V* originates from both the spatial distribution of photoexcited electrons and holes in the sample. More specifically, if we separate the contribution from electron and hole distribution on the electron-induced charges ($Q_{e-induced}$) and hole-induced charges ($Q_{h-induced}$) in the SPV probe, respectively, we have:

$$Q_{e-induced}=\frac{{I_{Pump}\cdot\lambda}_{Pump}}{hc}\cdot\left( 1-e^{{-\alpha}_{Pump}\cdot d} \right)\cdot E_{e}\left( I_{Pump} \right)\cdot k_{e}\left( I_{Pump} \right) (S12)$$

$$Q_{h-induced}=\frac{{I_{Pump}\cdot\lambda}_{Pump}}{hc}\cdot\left( 1-e^{{-\alpha}_{Pump}\cdot d} \right)\cdot E_{h}\left( I_{Pump} \right)\cdot k_{h}\left( I_{Pump} \right) (S11)$$

$$V=\left| Q_{h-induced}-Q_{e-induced} \right|{\cdot A}_{Q\to V} (S13)$$

where, analogous to the parameter definitions in Equation S3, $E_{e}\left( I_{Pump} \right)$ and $E_{h}\left( I_{Pump} \right)$ represent the electron and hole accumulation factor, respectively. $k_{e}\left( I_{Pump} \right)$ and $k_{h}\left( I_{Pump} \right)$describe the ability of inducing holes and electrons in the SPV probe, respectively, originating from the electric field on the mica side established by a specific spatial distribution of electrons and holes in the sample, respectively. Therefore, a weaker accumulated-electron-/hole-induced electric field on the mica side would lead to smaller $k_{e}\left( I_{Pump} \right)$/$k_{h}\left( I_{Pump} \right)$.

For instance, as illustrated in Figure S8a, after photoexcitation, electrons and holes accumulate at different sides due to the build-in field in the sample. The accumulated holes in the sample can induce the electrons in the SPV probe ($Q_{h-induced}$), while the accumulated electrons in the sample forming an opposite electric field on the mica side inducing the opposite charges of holes ($Q_{e-induced}$) at the surface of SPV probe. The total induced charge (in the SPV probe) $Q_{induced}=\left| Q_{h-induced}-Q_{e-induced} \right|$. Therefore, a longer effective spatial distance between the overall distribution of electrons and holes in the sample can lead to a larger number of $Q_{induced}$ in the SPV probe, resulting from an increased $Q_{h-induced}$ [via increasing $k_{h}\left( I_{Pump} \right)$] and/or a decreased $Q_{e-induced}$ [via decreasing $k_{e}\left( I_{Pump} \right)$].

In this case, we can expect that $k_{e}\left( I_{Pump} \right)$ in defect-free system (Figure S8a) is smaller than that in perovskite suffering from electron traps (Figure S8b), because the spatially trapped electrons result in a relatively larger overall electron-induced electric field on the mica side. Consequently, the system with electron traps (Figure S8b) would induce a smaller $Q_{induced}$ due to the larger $Q_{e-induced}$, compared with the defect-free system (Figure S8a). In terms of the ‘push’ effect, the trapped electrons could be delocalised and subsequently be drifted or diffuse towards the left surface upon the IR ‘push’ beam as shown in Figure S8c, where the displacement of the de-trapped electrons leads to a decrease of $k_{e}\left( I_{Pump} \right)$ and resulted smaller $Q_{e-induced}$. This leads to larger $Q_{induced}$ in the SPV probe and results in an additional SPV signal, which is collected as PP-SPV signal ($\Delta V$). Analogous process will also occur for the trapped holes in the PVSK/HTL interface. And vice versa if electrons and holes accumulate in opposite sides due to the opposite built-in field direction in a p-i-n device.

Along the same vein, we propose that the trapped carriers localised at the top surface could not contribute to the $\Delta V$ if the top surface is not covered by an upper layer. As an example illustrated in Figure S9, the ‘push’ beam can still energetically release the trapped holes accumulating at the top surface back to the valence band, but the spatial distribution of overall holes remains unchanged as they still stay at the top surface, resulting in an unchanged $k_{h}\left( I_{Pump} \right)$ and consequent identical $Q_{h-induced}$. This leads to no contribution from trapped holes to the SPV signal. Note that here we assume both trapped and free holes have a similar capability to generate induced electrons in the SPV probe. Therefore, for perovskite without upper HTL deposition, under the optical ‘push’, only electrons de-trapped from bottom interface and within the bulk could be identified as $\Delta V$, resulting from the change of $Q_{e-induced}$ (as demonstrated in Figure S8). And vice versa if electrons and holes accumulate in opposite sides and holes are trapped in the HTL/PVSK interface in a ITO/HTL/PVSK sample.

Concerning the low density of traps within the bulk of perovskite material, we rationalise that in the sample without top CTL, the PP-SPV signal is dominated by the trapped carriers at the buried interface of perovskite. This is further confirmed by the scaling of signal magnitude with additional interfaces established, as shown in Figure S10.

**Figure S10. The PP-SPV signal collected from the layer stacks with different combinations of CTLs.** The PP-SPV signal in ITO/PVSK is negligible, and could be substantially increased when more CTLs are deposited.

**Figure S11.** (a) Pump-induced SPV and (b) quasi-steady-state PP-SPV from whole/half devices with conventional configuration. The intensity of ‘push’ is kept at 10600 mW cm^‒2^. All the curves in (b) are fitted by power law.

**Figure S12.** (a) Pump-induced SPV and (b) quasi-steady-state PP-SPV from whole/half devices with inverted configuration. The intensity of ‘push’ is kept at 20800 mW cm^‒2^. All the curves in (b) are fitted by power law.

**Figure S13.** Trapped carrier dynamics measured by transient PP-SPV for devices with (a) conventional configuration and (b) inverted configuration.

**Figure S14. Trap filling dynamics of samples with/without metal electrode.** PP-SPV transients measured from samples with (a) conventional device structure and (b) inverted device structure.

**Figure S15.** **Fluence dependence of pump-induced SPV in n-i-p samples with different function layer combinations.**

**Figure S16.** **Pump fluence dependence of the transient PP-SPV signals from half cells with different conventional configuration.** The trapped carrier dynamics in ITO/SnO_2_/PVSK/OAI/Spiro sample (a) before and (b) after normalisation. The trapped carrier dynamics in ITO/SnO_2_/PVSK/OAI sample (c) before and (d) after normalisation. The push fluence is kept at 1.91 mJ cm^‒2^ for all measurements. The fluence-independent trapped carrier dynamics suggests that trapped carriers in different film stacks behave individually without higher-order interaction.

**Figure S17. Rising time (*τ_rise_*) distribution of five n-i-p samples with/without upper layer deposition.** The *τ_rise_* is defined as the time taken to reach 90% of the signal’s full height.

**Figure S18.** **Fluence dependence of pump-induced SPV in p-i-n samples with different function layer combinations.**

**Figure S19.** **Pump fluence dependence of the transient PP-SPV signals from different combination of function layers assembled as inverted device architecture.** The trapped carrier dynamics in ITO/NiO*_x_*/SAM/PVSK/OAI/BCP/C_60_ sample (a) before and (b) after normalisation. The trapped carrier dynamics in ITO/NiO*_x_*/SAM/PVSK/OAI sample (c) before and (d) after normalisation. The push fluence is kept at 1.34 mJ cm^‒2^ for all measurements.

**Figure S20. *τ_rise_* distribution of five p-i-n sample with/without upper layer deposition.**

**Figure S21. *J*-*V* curves of electron and hole only devices.** The inserted equations are used for the extraction of trap density ($N_{t}$, based on identifying of trap-filled limit voltage $V_{TFL}$) and mobility ($\mu$, based on fitting the Child regime by Mott-Gurney Law). In the equations, $\varepsilon_{0}$ $\varepsilon$, $q$, $V_{BI}$, and $L$ represent vacuum permittivity, relative permittivity (set as 60), elementary charge, built-in voltage (set as 1 V), and perovskite thickness (800 nm), respectively. The extracted electron trap density and mobility of perovskite from an electron only device are 2.6×10^15^ cm^‒3^ (based on $V_{TFL}$ of 1.32 V) and 6.0×10^‒2^ cm^2^ V^‒1^ S^‒1^, respectively. The extracted hole trap density and mobility of perovskite from a hole only device are 2.8×10^15^ cm^‒3^ (based on $V_{TFL}$ of 1.46 V) and 1.5×10^‒2^ cm^2^ V^‒1^ S^‒1^, respectively. We raise the concern that the trap density estimated by SCLC measurement involves all electron or hole traps within bulk and surfaces of perovskite. In contrast, PP-SPV detects only trapped carriers that contribute to substantial non-radiative loss, specifically those that cannot undergo thermal de-trapping at device operational conditions and are active species at the corresponding interfaces (e.g., hole trap at HTL/PVSK interface and electron trap at ETL/PVSK interface).

**Supplementary Note 5: Drift-Diffusion Model**

We employ the drift-diffusion model to simulate semiconductor devices by solving coupled partial-differential equations.^[7]^ These equations allow us to calculate the time- and position-dependent electrostatic potential $V\left( x,t \right)$ through the Poisson equation, as well as the densities of free electrons $n\left( x,t \right)$ and holes $p\left( x,t \right)$. The model captures the drift caused by the electric field (via the gradient of the electric potential) and the diffusion arising from concentration gradients of electrons and holes. Additionally, it incorporates recombination processes. The complete set of equations is as follows:

$$\begin{aligned} C \cdot\frac{\partial u}{\partial t}=x^{-m}\frac{\partial}{\partial x}{(x}^{m}f)+s \#\left( S14 \right) \end{aligned}$$

In which:

$$\begin{aligned} u=\left[ \begin{aligned} \begin{aligned} \begin{matrix} V \\ n \\ p \end{matrix} \end{aligned} \end{aligned} \right] C=\left[ \begin{aligned} \begin{aligned} \begin{matrix} 0 \\ 1 \\ 1 \end{matrix} \end{aligned} \end{aligned} \right] f=\left[ \begin{aligned} \begin{aligned} \begin{matrix} \frac{\epsilon_{r}\left( x \right)}{\epsilon_{max}}\frac{dV}{dx} \\ -j_{n} \\ -j_{p} \end{matrix} \end{aligned} \end{aligned} \right] s=\left[ \begin{aligned} \begin{aligned} \begin{matrix} \frac{q}{\epsilon_{max}\epsilon_{0}}\left( p\left( x,t \right)-n\left( x,t \right) \right) \\ g_{n}\left( x,t \right)-r_{n} \left( x,t \right) \\ g_{p}\left( x,t \right)-r_{p}\left( x,t \right) \end{matrix} \end{aligned} \end{aligned} \right]\#\left( S15 \right) \end{aligned}$$

where, $\epsilon_{r}\left( x \right)$ is relative permittivity; $q$ is the unit charge; $g_{n},g_{p}$ are the carrier generation rate of electrons and holes under laser excitation, respectively, (the spatial dependence origin from Beer-Lambert law); $j_{n},j_{p}$ denote electron and hole flux, respectively; and $r_{n},r_{p}$ denote recombination rates for electrons, holes, respectively.

To accurately estimate the dynamics of trapped carriers at the interface, we considered the input flux from drift and diffusion terms contribute to two output pathways (i.e., the charge extraction and trapping terms). Consequently, the presence of trapped carriers indirectly influences the extraction path (Equations S16 and S17).

$$-D_{n}\frac{dn_{b}}{dx}+\mu_{n}\cdot n_{b}\cdot E_{b}{=k}_{en}\cdot n_{b}{+ k}_{tn}\cdot n_{b}\cdot\left( N_{tn}-n_{trap} \right) \left( S16 \right)$$

$$-D_{p}\frac{dp_{b}}{dx}+\mu_{p}\cdot p_{b}\cdot E_{b}{=k}_{ep}\cdot p_{b}{+ k}_{tp}\cdot p_{b}\cdot\left( N_{tp}-p_{trap} \right) \left( S17 \right)$$

where, $n_{trap},p_{trap}$ is the trapped electron and hole density and $N_{tn},N_{tp}$ is the electron trap and hole trap state density; $n_{b}, p_{b}$ is the electron and hole density at boundary; $E_{b}$ is the electrical field at boundary; $k_{tn}, k_{tp}$ is the electron and hole trapping rate; $k_{en},k_{ep}$ is the electron and hole extraction rate at boundary, $D_{n},D_{p}$ is the diffusion coefficient of electrons and holes in the perovskite; $\mu_{n}$, $\mu_{p}$ is the mobility of electrons and holes in the perovskite.

Based on the Shockley–Read–Hall rate equation, we can calculate the concentration of trapped carriers at the ETL and HTL interfaces in real time and determine the variation in trapped carrier concentration using the following equations S18–S19:

$$\begin{aligned} \frac{{dn}_{trap}}{dt}=k_{tn}\cdot n_{b}\cdot\left( N_{tn}-n_{trap} \right)\#\left( S18 \right) \end{aligned}$$

$$\begin{aligned} \frac{{dp}_{trap}}{dt}=k_{tp}\cdot p_{b}\cdot\left( N_{tp}-p_{trap} \right)\#\left( S19 \right) \end{aligned}$$

Equations S20–S24 show the generalised form for boundary condition of two types of devices. For $V\left( x,t \right)$, we applied Dirichlet boundary condition since the electrode of device has fixed potential after connection. $V_{r}$, $V_{l}$, $V_{app}$, and $V_{bi}$ are the potential of right electrode, the potential of left electrode, the applied bias, and the built-in potential difference, respectively. For $\frac{n\left( x,t \right)}{p\left( x,t \right)}$, we applied Neumann boundary conditions.

A general form for all boundary condition is:

$$\begin{aligned} p\left( x,t,u \right)+q\left( x,t \right)f\left( x,t,u,\frac{du}{dx} \right)=0 \#\left( S20 \right) \end{aligned}$$

For conventional device (ETL/PVSK/HTL):

$$\begin{aligned} \begin{aligned} u=\left[ \begin{aligned} \begin{matrix} V \\ n \\ p \end{matrix} \end{aligned} \right] p_{l}=\left[ \begin{aligned} \begin{aligned} \begin{matrix} -V_{l} \\ k_{en}\cdot n_{b}{+ k}_{tn}\cdot n_{b}\cdot\left( N_{tn}-n_{trap} \right) \\ 0 \end{matrix} \end{aligned} \end{aligned} \right] ql= \left[ \begin{aligned} \begin{matrix} 0 \\ 1 \\ 1 \end{matrix} \end{aligned} \right]\#\left( S21 \right) \end{aligned} \end{aligned}$$

$$\begin{aligned} p_{r}=\left[ \begin{aligned} \begin{matrix} -V_{r}+V_{bi}-V_{app}-V_{res} \\ 0 \\ k_{ep}\cdot p_{b}{+ k}_{tp}\cdot p_{b}\cdot\left( N_{tp}-p_{trap} \right) \end{matrix} \end{aligned} \right] qr= \left[ \begin{aligned} \begin{matrix} 0 \\ 1 \\ 1 \end{matrix} \end{aligned} \right]\#\left( S22 \right) \end{aligned}$$

For inverted device (HTL/PVSK/ETL):

$$\begin{aligned} \begin{aligned} u=\left[ \begin{aligned} \begin{matrix} V \\ n \\ p \end{matrix} \end{aligned} \right] p_{l}=\left[ \begin{aligned} \begin{aligned} \begin{matrix} -V_{l} \\ 0 \\ k_{ep}\cdot p_{b}{+ k}_{tp}\cdot p_{b}\cdot\left( N_{tp}-p_{trap} \right) \end{matrix} \end{aligned} \end{aligned} \right] ql= \left[ \begin{aligned} \begin{matrix} 0 \\ 1 \\ 1 \end{matrix} \end{aligned} \right]\#\left( S23 \right) \end{aligned} \end{aligned}$$

$$\begin{aligned} p_{r}=\left[ \begin{aligned} \begin{matrix} -V_{r}+V_{bi}-V_{app}-V_{res} \\ k_{en}\cdot n_{b}{+ k}_{tn}\cdot n_{b}\cdot\left( N_{tn}-n_{trap} \right) \\ 0 \end{matrix} \end{aligned} \right] qr= \left[ \begin{aligned} \begin{matrix} 0 \\ 1 \\ 1 \end{matrix} \end{aligned} \right]\#\left( S24 \right) \end{aligned}$$

Through this approach, at the boundary (ETL or HTL interfaces) we can simultaneously obtain the time-dependent free carrier concentration as well as the trapped carrier concentrations. According to Supplementary Note 3, the IR activated PP-SPV signal ($\Delta V/V$) is originated from the trapped carriers localised at interfaces, as long as the release of these carriers by the IR push undergo spatial redistribution and modify the photovoltage signal. For samples covered by an upper layer, the PP-SPV signal corresponds to the de-trapped carrier at both the ETL and HTL interfaces. Therefore, we simulated the concentration evolution of trapped carrier at both interfaces through Driftfusion model which coincides with measured $\Delta V/V$. Similarly, for samples without a top layer, the PP-SPV signal corresponds to the de-trapped carrier density only at the bottom interface. Therefore, we simulated the concentration evolution of trapped carrier at the bottom interface through Driftfusion model which is consistent with measured $\Delta V/V$.

The parameters used for Equation S21–S24 are summarised in Table S2.

**Figure S22. Morphology images of perovskite (a) before (ITO/NiO_x_/SAM/PVSK/OAI) and (b) after PVP passivation (ITO/NiO_x_/SAM/PVP/PVSK/OAI) obtained by scanning electron microscopy.**

**Figure S23. X-ray diffraction of perovskite films with/without PVP passivation.** The similar full-width at half-maximum of the main diffraction peaks indicates comparable crystal sizes in both samples, consistent with the minimal variation of grain size visualised in SEM figures (Figure S22). This result also suggests the crystallinity is unaffected by the bottom interface passivation.

**Table S1. Summary of photovoltaic parameters of conventional and inverted PeSCs.**

|  | *V_oc_* (V) | *J_sc_* (mA cm^‒2^) | FF (%) | PCE (%) |
| --- | --- | --- | --- | --- |
| Conventional - forward | 1.09 | 24.5 | 63.7 | 17.0 |
| Conventional - reverse | 1.13 | 24.6 | 75.1 | 21.0 |
| Inverted - forward | 1.09 | 24.4 | 67.7 | 18.0 |
| Inverted - reverse | 1.12 | 24.5 | 74.9 | 20.5 |

**Table S2. The parameters in drift-diffusion model used for transient PP-SPV simulation.**

| Parameter | Regular device | Inverted device | Description | Parameter source |
| --- | --- | --- | --- | --- |
| *T* (K) | 300 | 300 | Temperature | Empirical constants based on Ref [7] |
| *B* (cm^3^ s^-1^) | 1×10^‒12^ | 1×10^‒12^ | Radiative recombination rate constant |  |
| *N*_CB_, *N*_VB_ (cm^-3^) | 1×10^18^ | 1×10^18^ | Effective density of states |  |
| $k_{tn}$, $k_{tp}$ (cm^3^ s^-1^) | 1×10^‒12^ | 1×10^‒12^ | Electron and hole trapping rate constant |  |
| *n*_t0_ (cm^-3^) | 1×10^15^ | 1×10^15^ | Initial carrier density |  |
| $\mu_{n}$ (cm^2^ V^-1^ s^-1^) | 1.5×10^‒2^ | 1.1×10^‒2^ | Electron mobility | Extracted from measurement in Figure S21 |
| $\mu_{p}$ (cm^2^ V^-1^ s^-1^) | 1.5×10^‒2^ | 1.1×10^‒2^ | Hole mobility |  |
| $N_{tn}$ (cm^-3^) | 1×10^15^ | 1×10^15^ | Electron trap density | Simulation results |
| $N_{tp}$ (cm^-3^) | 2×10^15^ | 2×10^15^ | Hole trap density |  |
| $k_{en}$ (s^-1^) | 1×10^6^ | 1×10^6^ | Electron extraction rate |  |
| $k_{ep}$ (s^-1^) | 1×10^6^ | 1×10^6^ | Hole extraction rate |  |

**Reference**

[1] Y. Liu, J. Wang, N. Zhu, W. Liu, C. Wu, C. Liu, L. Xiao, Z. Chen, S. Wang, *Opt. Lett.* **2019**, *44*, 3474.

[2] B. P. Carwithen, T. R. Hopper, Z. Ge, N. Mondal, T. Wang, R. Mazlumian, X. Zheng, F. Krieg, F. Montanarella, G. Nedelcu, M. Kroll, M. A. Siguan, J. M. Frost, K. Leo, Y. Vaynzof, M. I. Bodnarchuk, M. V. Kovalenko, A. A. Bakulin, *ACS Nano* **2023**, *17*, 7, 6638–6648.

[3] J. Pan, Z. Chen, T. Zhang, B. Hu, H. Ning, Z. Meng, Z. Su, D. Nodari, W. Xu, G. Min, M. Chen, X. Liu, N. Gasparini, S. A. Haque, P. R. F. Barnes, F. Gao, A. A. Bakulin, *Nat. Commun.* 2023, *14*, 8000.

[4] Z. Xie, S. Sun, Y. Yan, L. Zhang, R. Hou, F. Tian, G. G. Qin, *J. Condens. Matter Phys.* **2017**, *29*, 245702.

[5] A. Musiienko, D. R. Ceratti, J. Pipek, M. Brynza, H. Elhadidy, E. Belas, M. Betušiak, G. Delport, P. Praus, *Adv. Funct. Mater.* 2021, *31*, 2104467.

[6] Z. Meng, E. Pastor, S. Selim, H. Ning, M. Maimaris, A. Kafizas, J. R. Durrant, A. A. Bakulin, *J. Am. Chem. Soc.* 2023, 145, *32*, 17700.

[7] P. Calado, I. Gelmetti, B. Hilton, M. Azzouzi, J. Nelson, P. R. F. Barnes, *J. Comput. Electron* **2022**, *21*, 960.
